# Young-Onset Carcinogenesis – The Potential Impact of Perinatal and Early Life Metabolic Influences on the Epigenome

**DOI:** 10.3389/fonc.2021.653289

**Published:** 2021-04-29

**Authors:** Savio George Barreto, Stephen J. Pandol

**Affiliations:** ^1^ Division of Surgery and Perioperative Medicine, Flinders Medical Center, Adelaide, SA, Australia; ^2^ College of Medicine and Public Health, Flinders University, Los Angeles, SA, Australia; ^3^ Division of Digestive and Liver Diseases, Cedars-Sinai Medical Center, Los Angeles, CA, United States

**Keywords:** survival, incidence, hypothesis, epigenetics, acetyl-CoA

## Abstract

The last decade has witnessed a significant rise in cancers in young adults. This spectrum of solid organ cancers occurring in individuals under the age of 40 years (some reports extending the age-group to <50 years) in whom aetiology of cancer cannot be traced back to pre-existing familial cancer syndromes, is referred to as termed young-, or early- onset cancers. The underlying causes for young-onset carcinogenesis have remained speculative. We recently proposed a hypothesis to explain the causation of this entity. We propose that the risk for young-onset cancer begins in the perinatal period as a result of the exposure of the foetus to stressors, including maternal malnutrition, smoking or alcohol, with the consequent epigenomic events triggered to help the foetus cope/adapt. Exposure to the same stressors, early in the life of that individual, facilitates a re-activation of these ‘responses designed to be protective’ but ultimately resulting in a loss of regulation at a metabolic and/or genetic level culminating in the evolution of the neoplastic process. In this manuscript, we will provide a rationale for this hypothesis and present evidence to further support it by clarifying the pathways involved, including elucidating a role for Acetyl-CoA and its effect on the epigenome. We present strategies and experimental models that can be used to test the hypothesis. We believe that a concerted effort by experts in different, but complementary fields, such as epidemiology, genetics, and epigenetics united towards the common goal of deciphering the underlying cause for young-onset cancers is the urgent need. Such efforts might serve to prove, or disprove, the presented hypothesis. However, the more important aim is to develop strategies to reverse the disturbing trend of the rise in young-onset cancers.

## Introduction

The term young-, or early- onset cancers (1, 2) encompasses a spectrum of solid organ cancers occurring in individuals under the age of 40 years [although some authors extend the upper limit of the age-group to include those <50 years (3)] in whom the aetiology of their cancers cannot be traced back to pre-existing familial cancer syndromes (4–6). This entity has been reported to affect nearly every solid organ including the pancreas (7), breast (8), ovarian (5), oesophageal (9), colorectal (10, 11), and gastric (12) cancer, amongst others. The large number of reports on the rising incidence of this entity from around the world (3, 13–15) have left oncologists questioning the cause for the cancers and their disturbing trend. At the present time, the only strategy to combat the rising incidence of young-onset cancers is to reduce the age of screening (15).

Recent data on Adolescent and Young Adult (AYA) cancers from the American Cancer Society (16) has indicated that while the incidence of young-onset cancers has increased at the rate of 1% annually in adolescents and 0.4-1.1% annually in women aged 20-39 years, the rising trend is not uniform across the board for all cancers but demonstrates a characteristic pattern in terms of organ involved based on age distribution (15-9years vs 20-29years vs 30-39years), gender and race based on data from the United States. For instance, adolescents (15- to 19-year-olds) have a higher burden of acute lymphocytic leukaemia and lymphoma, while 20–39-year-old individuals have a higher incidence of solid organ cancers (17). The rise in the incidence rates of cancers in females aged 20-39 years has largely been driven by increases in breast cancer, as well as thyroid cancer and melanoma of the skin (17). A significant increase in colorectal, endometrial, renal and female breast cancers were noted in the decade 2007-2016 in all adults between the ages 20-39years.

**Figure 1:**
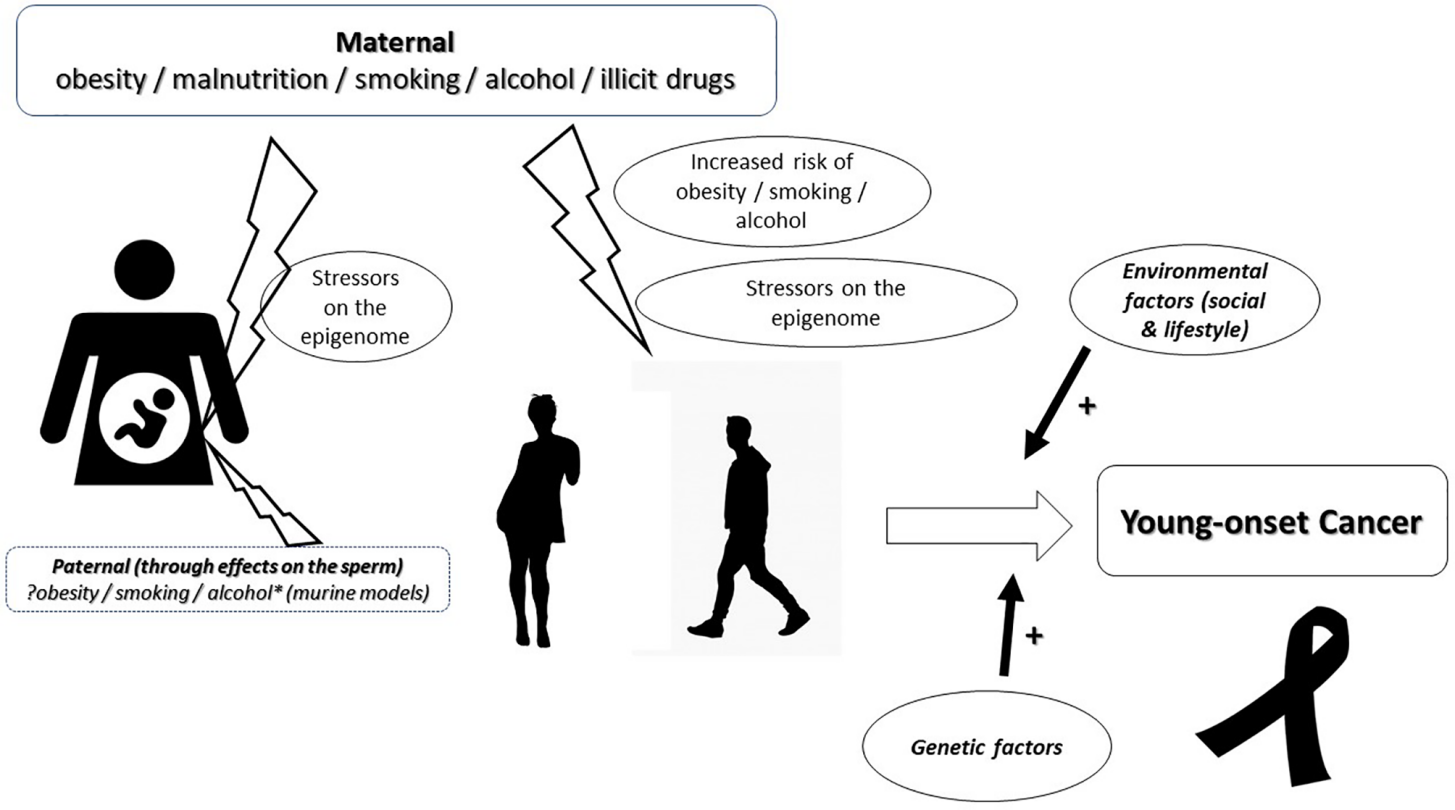
Diagrammatic representation of the hypothesis for the causation of young-onset carcinogenesis. The risk for young-onset cancer begins *in utero* with exposure of the foetus to maternal, stressors. The possibility of a paternal contribution through the damaging effects of stressors on the sperm are considered and warrant consideration. Exposure to the same stressors, during adolescence or young adulthood, facilitates a re-activation of these ‘responses designed to be protective’ but ultimately resulting in a loss of regulation at a metabolic and/or genetic level culminating in the evolution of the neoplastic process as a result of a cumulative effect with environmental and other genetic factors.

Exposure to antibiotics in early life, rising incidence of obesity, cigarette smoking, impact of the gut microbiome, and variations in MMR genes and MSI are some factors postulated to play a role in young-onset carcinogenesis (4, 15). However, taking into consideration the ‘two-hit’ hypothesis of carcinogenesis postulated by Knudson (18) and their timing in sporadic cancers based on the Cancer generative model (19), more recently, we proposed a hypothesis for young-onset carcinogenesis. We hypothesize that the risk for young-onset cancer begins in the perinatal period following foetal exposure to stressors, including maternal malnutrition, smoking or alcohol, with the consequent triggering of epigenomic events aimed at helping the foetus cope/adapt to these stressors. Exposure to the same stressors, early in that individual’s life, reactivates these ‘responses designed to be protective’ but ultimately resulting in a loss of regulation at a metabolic and/or genetic level culminating in neoplastic evolution (**Figure 1**). In summary, we postulate that an ‘*in utero’* insult to the foetus speeds up, or leads to, the ‘first hit’. The second hit would then be the result of processes occurring in childhood and adolescence.

In this manuscript, we will provide a rationale for this hypothesis and present evidence to further support it by clarifying the pathways involved. We believe this will help foster International collaboration to focus on combatting potentially correctable underlying mechanisms to help reduce the overall incidence of young-onset cancers.

## Rationale for the Hypothesis

Hereditary cancer predisposition syndromes account for 5-10% of all cancers that develop in individuals who have inherited a genetic mutation conferring heightened susceptibility to specific cancers (20). The various classes of genes involved in Hereditary cancer predisposition include tumour suppressor genes, oncogenes, genes encoding proteins involved in DNA repair and cell cycle control, and genes involved in stimulating the angiogenic pathway, or genes involved in carcinogen metabolism (21). The hypothesis put forth by us is intended to explain young-onset carcinogenesis amongst individuals in whom the aetiology of their cancers cannot be traced back to pre-existing familial cancer syndromes (4–6).

Earlier this year, Lahouel et al. (19) published a data-driven, mathematical model of the process of tumour evolution taking into consideration the fitness advantages for driver genes and carrying capacity (19) to plot the timeline of mutational events in driver genes. The authors drew on their vast experience of decades of cancer research to prepare the model that accounts for the 3 mechanisms that confer fitness advantage, namely, cell fate, cell survival and genome maintenance, and total number of clonal somatic mutations accumulated in a cell lineage. Using this model, they shed light on the evolutionary dynamics of cancer by suggesting a generalized early onset of tumorigenesis followed by slow mutational waves. This was in stark contrast to previous thought processes. They noted the ‘first hit’ to occur at a median age of 14.4 year for colon cancer and 14.6 years for pancreatic cancer, with the full development of malignancy taking on average another 50 years. In young-onset cancers, we can postulate an acceleration of events in terms of the timing of both ‘hits’. However, it is difficult to accept that an individual would be exposed to the proposed risk factors within the first couple of decades of life to an extent that would induce mutations and cancer by the age of 40 years. The ‘Developmental Origins of Health and Disease (DOHaD)’ (22) hypothesis, postulated to describe the incidence of chronic diseases in adult life highlighted the significance of exposure of the developing foetus to a hostile (nutritional deprivation or excess, chemical exposure, or infections) environment in relation to an increased disease risk. These *in utero* insults can trigger epigenetic and hormonal modifications designed to permit the growing foetus to adapt and survive. Would it be possible then that the effects of exposure to similar risk factors in early childhood and adolescence could trigger epigenetic modifications resulting in a premature activation of driver mutations (the ‘second hit’) rendering the young adult ‘at risk’ of developing a cancer? (1) The theory of ‘stress-induced’ mutagenesis (23) would certainly lend support to this.

Traditional dogma would dictate that cancer is related to the growth of the organ based on an appreciation that the number of stem cell divisions occurring in a tissue during life might dictate cancer risk (24). However, this is not entirely true, especially in young-onset cancers where there is clearly demonstrable variations in the organ involved depending on the individual’s age, gender, and even race (16). This would point to importance of other factors, ‘extrinsic’ to the gene (25), notably, metabolic [including mitochondrial (26)] and epigenomic factors in determining the self-renewal capacity of the cell (27).

To investigate the hypothesis, in the absence of direct causal evidence for young-onset cancer, it is imperative that we determine if there exists a chain of evidence linking perinatal metabolic influences (including, but not limited to, alcohol, smoking, illicit drugs, over-, as well as, under nutrition) to similar influences in adolescence and early adulthood. We would then need to find an increased risk of early-onset carcinogenesis in this cohort of individuals. This approach would allow us an opportunity to investigate the potential role of epigenetic mechanisms and other factors influencing the changes in these individuals.

## Metabolic Influences in the Perinatal Period and the Risk on the Offspring

We are in the midst of a global obesity pandemic (28) that correlates with a high prevalence of maternal obesity (29). The last few decades have witnessed a significant rise in childhood and adolescent obesity (30). Maternal pre-pregnancy obesity and weight gain during pregnancy are risk factors for obesity in the offspring (31). Epidemiological evidence (32) supports the foetal overnutrition hypothesis that proposes that greater maternal adiposity results in increased obesity throughout life in the offspring (33) - the effect extending even up to the age of 21 years (34). In fact, nutrition, as a whole, is a significant factor in foetal programming in the context of the DOHaD hypothesis (22). Evidence from individuals exposed to famines has presented a unique glimpse into the risk of their offspring being overweight and obese (35, 36). Environmental and genetic factors alone cannot explain the risk of metabolic risk factors in the offspring of affected individuals (37). There is compelling evidence to support the role of maternal nutrition on foetal metabolic programming mediated *via* epigenetic modifications (38, 39).

Alcohol use during pregnancy is established as a risk factor for adverse outcomes to the mother and foetus with the global prevalence of alcohol use during pregnancy estimated to be 9·8% (95% CI 8·9–11·1) (40). Alcohol consumption during pregnancy is not restricted to a specific race (41), although some ethnic backgrounds have been reported to be at a higher risk of FAS and FASD (42). The significance of binge drinking in these situations remains a concern (43). High-intensity drinking remains a cause for concern as these behaviours persist through to the adult years (44). Longitudinal studies have clearly demonstrated that foetal alcohol exposure was associated with alcohol problems in early adolescence (even after controlling for family history of alcoholism, prenatal nicotine exposure, parenting style, current parental drinking, household stress, and self-esteem) (45) and young adulthood (46). Thus, although there has been a demonstrable overall reduction in adolescent drinking worldwide (47), this does not hold true for the offspring of mothers who used alcohol during pregnancy.

Similarly, tobacco and illicit substance use (up to 15%) is a concern amongst women in the reproductive age group and pregnant women (48, 49). The risk of misuse amongst offspring has been confirmed (50) with the possibility of genetic transmission of the risk being touted as an underlying mechanism.

The three factors proposed for epigenetic events serve as examples. As reviewed by Dai et al. (51) epigenetic regulation can occur not only on DNA, but also on RNA and nuclear histones. Furthermore, there are several potential epigenetic alterations that have been identified in addition to acetylation and methylation. These include lactylation, succinylation, homocysteinylation, and butylation, among others (51). These findings indicate that the nutritional state, microbiome, inflammation through changes in metabolite availability, and the redox state (in addition to expression of enzymes needed) can have epigenetic effects. **Table 1** lists some of the stressors and the proven mechanisms by which they alter the epigenome (52–65). Further, because the exposures in adulthood likely differ from the *in utero* environment, the secondary events during adulthood can differ biochemically. This raises the possibility that dissimilar, but interacting, epigenetic events could promote carcinogenesis.

**Table 1:** Factors listed as examples of stressors in the presented hypothesis and the mechanisms by which they alter the epigenome.

Metabolic Factor	Epigenetic modification	References
***Malnutrition***		
Methionine restriction Folate deficiency	DNA/histone methylation through the action of intracellular SAM	(52–54)
High fat diet	Histone acetylation through the action of Acyl-CoA DNA methylation	(55) (56, 57)
High glucose intake	Histone acetylation through the action of Acetyl-CoA	(58)
***Smoking***	Histone acetylation through the effects of acrolein	(59)
	Altered DNA methylation through the effects of PAH and	(60)
	perfluoroalkyl compounds, especially PFOA	(61)
***Alcohol***	Histone acetylation through the action of Acetyl-CoA	(62–64)
***Microbiome***	Histone crotonylation through histone deacetylases	(65)

These modifications, in turn, are known to play a role in carcinogenesis (DNA, deoxyribonucleic acid; PAH, polycyclic aromatic hydrocarbons; PFOA, perfluorooctanoate; SAM, s-adenosylmethionine).

In summary, children born to mothers with metabolic risk factors (malnutrition, smoking and/or alcohol exposure) are at an increased risk of, or susceptible to, similar risk factors in their own lifetimes. Additionally, these risks are noted to occur early in the lives of the offspring.

## Metabolic Influences on Spermatogenesis – Is There a Paternal Contributory Influence?

A hitherto underappreciated contribution to perinatal stressors includes the effects of the metabolic influences (nutrition, alcohol and smoking, amongst others) on spermatogenesis and the sperm. This is likely due to the belief that these stressors would deleteriously affect spermatogenesis to a point of inducing infertility (66, 67), and hence be inconsequential in terms of affecting the offspring. Besides, the Investigators in the Pregnancy And Childhood Epigenetics Consortium PACE have recently reported the lack of an effect of paternal BMI on their offspring-blood DNA methylation (68). However, there is evidence that smoking, for instance, may damage the sperm not to the extent to induce infertility (69, 70), but as a result of oxidative stress led to the formation of DNA and protein adducts in spermatozoa (71). Perrin et al. (72) determined that the formation of these DNA adducts in sperm cells may give rise to carcinogenic damage and prezygotic DNA damage that can be transmitted to offspring. This has been found by other investigators to assume the form of paternally derived BPDE‐DNA adducts in preimplantation embryos (73).

Alcohol, too, alters epigenetic mechanisms in the sperm (74) in murine experimental models including DNA methylation, chromatin modifications, and non-coding RNAs (74). These effects have been noted to be transferable across generations (74).

The high risk of foetal abortion or developmental disorders amongst fertile men exposed to stressors such as smoking (69) would further reduce the impact of paternal contributory factors towards young-onset carcinogenesis. However, this remains an area that warrants conclusive evidence to refute its contribution altogether.

## Childhood Obesity and Young-Onset Cancers

Observational studies have noted a higher incidence of adult cancers in individuals who were obese during childhood. The incidence of endometroid adenocarcinoma was found to be significantly higher in girls who were obese as children. A BMI z-score of 1.5 at the age of 7 years was associated with a had a hazard ratio=1.53 (95% confidence interval: 1.29-1.82) for endometroid adenocarcinoma compared to a z-score of 0 (75). Similarly, childhood BMI z-score at the age of 13 years was positively associated with a risk of colon cancer in adulthood (HR=1.09; 95% CI 1.04-1.14) (76). Being overweight and/or obese in adolescence is associated with a risk for colon cancer among both adult men (HR for overweight, 1.53; 95% confidence interval [CI], 1.28-1.84; HR for obesity, 1.54; 95% CI, 1.15-2.06; statistically significant from a BMI of 23.4 kg/m^2^) and women (HR for overweight, 1.54; 95% CI, 1.22-1.93; HR for obesity, 1.51; 95% CI, 0.89-2.57; significant from a BMI of 23.6 kg/m^2^). However, only obesity, but not being overweight in adolescence, was associated with a risk for rectal cancer amongst adult men (HR, 1.71; 95% CI, 1.11-2.65; significant from a BMI of 29.6 kg/m^2^) and women (HR, 2.03; 95% CI, 0.90-4.58; significant from a BMI of 30.6 kg/m^2^) (77).

A recent review of the studies using the Copenhagen School Health Records Register (78) found that childhood obesity was positively associated with risks of bladder (only late childhood), colon, endometrial, kidney, liver, oesophageal (only late childhood), ovarian, pancreatic (<70 years), prostate (only before childhood height adjustment) and thyroid cancer.

## Foetal Alcohol Spectrum Disorder (FASD) and Young-Onset Cancers

Himmelreich et al. (79) recently published the results of an anonymous, community-based health survey developed by them in adults with FASD. They analysed the data from 541 (out of 612) respondents who completed the survey. The ages of the respondents ranged from ≤16 to greater than 60 years, with the greatest number of individuals in the 16–40-year range. The sample was reasonably balanced by gender, with 52.8% females and 45.5% males, and including 0.8% “other” and 1.0% “rather not say”. Altogether, 47.8% of respondents were diagnosed with FAS and 17% with ARND while the remaining ~35% were diagnosed with pFAS, FAE, static encephalopathy PAE, and other disorders.

In this survey, 18 individuals (3.75% of respondents) reported having had some form of cancer (including cervical, Hodgkin’s lymphoma, liver, malignant melanoma, prostate, skin, thyroid, and embryonal rhabdomyosarcoma): five during childhood (<18 years), nine at 18–44 years, and four at 45–55 years. Most respondents who reported cancer were under 44 years of age leading to the frequency among these individuals being almost twice the prevalence in the general population.

Maternal alcohol consumption during pregnancy is associated with a significant risk of acute myeloid leukaemia in young children (80) prompting a call for primary prevention to reduce the risk (81).

In summary, children exposed to prenatal metabolic risk factors, through their mothers, are at a higher risk of cancer compared to the general population.

## Metabolic Effects on the Epigenome

Epigenetic mechanisms are essential for normal development and maintenance of tissue-specific gene expression patterns (82). However, patterns of DNA methylation and chromatin structure are altered in cancer (83). Epigenetic alterations are believed to be key initiating events in carcinogenesis (84). Zhang et al. (85) hypothesized a role for epigenetic mechanisms underlying the effect of maternal stress and associated sleeping disorders on their offspring which ultimately shape the immune system and gut health leading to an increased the risk for early-onset colorectal cancer.

The epigenome is susceptible to metabolic effects owing to metabolites serving the role of substrates for the generation of chromatin modifications (86). Chromatin is susceptible to alterations by covalent modifications on the histones by the actions of enzymes including DNMTs, HMTs and HATs. The rates of reaction of these enzymes are dependent on changes in substrate availability (86). Nutrient uptake by the cell, and its subsequent metabolism, results in the generation of key substrates, including, though not limited to, Acetyl-CoA and SAM. Levels of these substrates are thus regulated by nutrient availability (87). In murine models, maternal obesity has been found to induce epigenetic modifications in the foetus (88).

Obesity is a proven risk factor for cancer and cancer-related mortality (89). Calle et al. (90) interrogated the data of nearly 900,000 adults in the United States and noted that a BMI ≥40kg/m^2^ was associated with a higher cancer-related mortality for all cancers combined in addition to cancers at multiple specific sites. A state of chronic inflammation encountered in obese individuals has been touted to be one of the more significant drivers of obesity-related cancer (91). Nutrient excess, as well as deprivation, can influence the epigenome. Diet impacts tissue acyl-CoA and histone acetylation levels (55).

During nutrient deprivation, nuclear-cytoplasmic acetylation of proteins may decrease as aconsequence of both reduced availability of acetyl-CoA to promote KAT activation and rising levels of NAD+ or NAD+/NADH to promote sirtuin deacetylase activity (92). Reciprocally, in high nutrient situations, metabolic conditions inhibit sirtuins and high availability of acetyl-CoA could facilitate KAT activity (92). KATs act as central players in regulating transcription. They modify the levels of acetylation on target proteins and thereby allow cells to detect, respond, and directly meet their homeostatic needs (93).

Alcohol is metabolised by dehydrogenases to acetate and further into Acetyl-CoA (62) by the action of chromatin-bound ACSS2 (63) which contributes to histone acetylation (64). Compelling evidence from murine models indicates that gestational exposure to alcohol results in epigenetic alterations in the developing foetus (94). Cigarette smoke activates enzymes involved in DNA methylation and histone post-translational modifications, in addition to effects on non-coding RNA sequences (95). Prenatal cigarette smoking has been found to be associated with epigenetic modifications that persist in the offspring through to adolescence (96).

In addition to affecting epigenetics, metabolites also influence adult and embryonal stem cell behaviour through the action of Acetyl-CoA serving as an acyl donor for histone acetylation (97). However, we must remind the reader that epigenetic alterations are reversible (98). Cancer cells maintain an intrinsic plasticity that allows them to easily change their phenotype in response to new signals and possibly switch between cellular states (27).

## Increased Cancer Risk in the Offspring

It is noteworthy that the epigenome is susceptible to metabolic influences occurring at any period in an individual’s lifetime, commencing *in utero.* These effects on the epigenome not only last the entire lifetime of an individual (99), but have the potential to be transmitted across generations (74, 100), which is intriguing because it points to the observation that epigenomic changes “acquired” in one generation may be the inherited ‘first hit’ in the next, and potentially regardless of maternal behaviour.

It is important to clarify that the epigenome plays a role in the two-hit hypothesis either on its own (with evidence to support the role of DNA methylation as the second hit (101)) or by exerting its effect on tumour suppressor genes through the process of epigenetic silencing (102). Our hypothesis presented in this manuscript thus represents a variation from the long-held dogmas of carcinogenesis, namely, the inherited genetic susceptibility and acquired two-hit hypothesis (of Knudson) (18). The significance of the finding of germline mutations in cancer-predisposing genes, noted in 8.5% of children and adolescents with cancer, in the absence of a clear family history to predict the presence of an underlying predisposition syndrome (103), has yet to be clarified in the context of young-onset carcinogenesis. Metabolic (including mitochondrial) and genetic factors are not mutually exclusive. Acquired abnormalities in mitochondrial function could produce a type of vicious cycle where impaired mitochondrial energy production might initiate genome instability and mutability, which in turn could accelerate mitochondrial dysfunction and energy impairment in a cumulative way (104).

As highlighted above, offspring of mothers exposed to significant metabolic stressors during pregnancy (capable of inducing epigenetic modifications) are not only at an increased risk of similar metabolic stressors during their own lifetime with resultant effects on the epigenome, but would also be increasingly susceptible to premature activation of driver mutations by the concept referred to as ‘stress-induced mutagenesis’ (23). We can only deduce that the location of the resultant cancer would be strongly correlated (0.81) with the total number of divisions of the normal self-renewing cells maintaining that tissue’s homeostasis (24).

## Testing the Hypothesis and Its Implications

A deeper understanding of variations in young-onset cancer incidence based on subsites, age, sex and race might offer insights into the potential drivers of carcinogenesis and enable a testing of our proposed hypothesis.

There are various ways to test our hypothesis. While mathematical modelling using the Cancer Generative model (19) may help plot the time course of the first and second hit and support the hypothesis, another experimental design would involve a longitudinal observational study tracing pregnant females, their partners, and their offspring (including documenting their metabolic stressors such as nutrition, medical conditions, alcohol and smoking use), throughout their lives. Being able to document the actual incidence of cancer development would constitute the most objective strategy to prove, or disprove, the hypothesis. Frozen buffy coat specimens (105) collected from the mother, the male partner, the cord blood of the foetus (106), as well as from the offspring through various stages in their life, namely, childhood, adolescence and adulthood to study epigenetic modifications, including those related to acetyl-CoA could be a useful strategy to identify risks within that offspring, as well as, potential biomarkers for the early detection of cancer. The cohort of the patients studied by the American Cancer Society (90) that includes patients with an already proven increased risk of cancer mortality associated with overweight and obesity could be approached to test the hypotheses presented in this manuscript. Blood specimens, with or without specimens of saliva, could be collected from these individuals and assessed for abnormalities in their epigenome. Another cohort that can be approached to test this hypothesis are those patients with familial clustering, in whom inherited genetic susceptibility has been ruled out. Two such cohorts have been identified in Scandinavia (107, 108). The cohort of patients with breast cancer in the study by Heikkinen et al. (107) is a perfect example of a testable study group.

We accept that despite all these efforts, it may yet remain a challenge to prove cause and effect. Longitudinal studies with biochemical measurements following up children through to adulthood may show associations. These associations could be tested in preclinical models to prove causation and even elucidate the underlying mechanisms (109).

Data management and analysis of patients with young-onset cancers using artificial intelligence, machine learning and natural language processing addressing specific areas, especially genetics and environmental data (medical and lifestyle, social and commercial) will help define prediction modelling in terms of developing a framework focussing on early detection of these cancers.

However, the overarching question is, “Do we need to prove this hypothesis to commence interventions to reduce the risk of cancer in the offspring at risk?” The evidence to support the hypothesis is compelling. More importantly, the hypothesis raises issues that warrant urgent attention irrespective of the risk of carcinogenesis. Maternal, paternal, and childhood malnutrition (110), perinatal smoking (111) and alcohol abuse (40), teenage alcohol abuse (112) and smoking (113) remain major global health issues that need stronger preventative as well as remedial strategies to be laid down. Equally relevant is the fact that these stressors are inter-related. For instance, maternal smoking during pregnancy has been found to be associated not only with obesity in their own children (114), but even their grandchildren (115). The information generated from a better understanding of the timeline of carcinogenesis, would also serve to guide International Collaborative Initiatives, such as the CONCORD programme (116), to suggest recommendations on cancer surveillance for young adults who are deemed ‘at risk’. In addition, there is evidence to support the role of simple strategies such as dietary interventions, including caloric restriction, intermittent fasting or time-restricted feeding, in terms of improving the patients’ overall metabolic profiles (i.e., reduced body weight, improved glucose homeostasis) (37).

## Conclusion

Herein we propose a novel evidence-backed hypothesis for young-onset carcinogenesis. The implications of this hypothesis are significant and effort is necessary to investigate it to confirm, or refute, its veracity. We hope this hypothesis will serve as the starting point for directed research into the growing burden of young-onset carcinogenesis. The possibility that the stressors described in this manuscript could affect not only sperm quality (including density, motility, morphology and viability), but also its genetic and epigenetic content warrants consideration. The manuscript represents a call for action from various groups to work together to reduce the increasing burden of young-onset carcinogenesis.

## Data Availability Statement

The original contributions presented in the study are included in the article/supplementary material. Further inquiries can be directed to the corresponding authors.

## Author Contributions 

SB: Conceptualization and design, literature search, drafting the manuscript, final approval. SP: Design of the study, literature search, critical revision of manuscript, final approval. All authors contributed to the article and approved the submitted version.

## Funding

Our research was supported as follows: ***SP support*** - US NIH: R01 AA024464, P01 DK098108, P50 AA0119991, U01 DK108314. US DoD: W81XWH1910888.

## Conflict of Interest

The authors declare that the research was conducted in the absence of any commercial or financial relationships that could be construed as a potential conflict of interest.

## Abbreviations

ACSS2, acetyl-Co-A synthase-2; ARND, Alcohol-related neurodevelopmental disorder; BMI, body mass index; BPDE1, Benzo(a)pyrene diol epoxide 1; DNA, Deoxyribonucleic acid; DNMT, DNA methyltransferase; DOHaD, Developmental Origins of Health and Disease; FAE, Foetal alcohol effects; FAS, Foetal alcohol syndrome; FASD, Foetal alcohol spectrum disorder; HAT, histone acetyltransferase; HMT, histone methyltransferase; HR, hazard ratio; KAT, lysine acetyltransferases; MMR, Mismatch repair genes; MSI, Microsatellite instability; NAD, Nicotinamide adenine dinucleotide; NADH, Nicotinamide adenine dinucleotide + hydrogen; PAE, Prenatal alcohol exposure; SAM, S-adenosylmethionine.
